# The Municipalisation of All Hospitals

**Published:** 1902-12-06

**Authors:** 


### The Institutional Workshop.

### THE MUNICIPALISATION OF ALL HOSPITALS.

The question of what is termed the municipalisation of hospitals has popped up again, as seen from the report in our columns last week, and we think it well to once more dispose of this impracticable suggestion. At its birth a nation might possibly nowadays provide the community with hospitals through the municipal authorities. In such an event the State at the centre would have to set up and maintain great hospitals to which well organised medical schools must be attached in affiliation with a central university, where medical students could be educated as skilled medical practitioners. Under a scheme like that suggested a municipal hospital would really provide a house of recovery for those members of the community who chose to use it when ill or when overtaken by accident. It could, however, form no part of a system of hospitals on which the community could depend for the training of its doctors, and we take it that in practice the amount of hospital accommodation required at the hands of the municipality would be relatively small in the case of a new community, where everybody would have suitable accommodation in his own dwelling for a non-infectious case of illness. It is probable, too, that such a hospital would be little used, and that from this circumstance its efficiency would gradually diminish; nor is it likely that the general interest taken in a municipal hospital so constituted would be great.

In an old country like England the proposal to municipalise all the hospitals shows that the authors of this idea have never studied the question in any accurate sense at all. In the first place medical education must be carried on within the hospitals on a system which will guarantee to the population of these islands an adequate number of capable men and women for the treatment and cure of the sick. Our system of medical education, faulty and defective as it may be, would be utterly destroyed by the municipalisation of all the hospitals. It is not likely that municipal authorities will have the courage to demand of the ratepayer appliances, buildings and appurtenances of a great medical school or schools, not for their own immediate benefit, but for the benefit of the nation at large. It is even doubtful whether clinical instruction would be even permitted in municipal hospitals. To those who have studied the subject in every aspect, and with intelligence, it must be apparent that it is necessary that medical education and medical relief must go hand in hand in order that the interests of the public and patients alike may be secured and safeguarded. The instruction in principles and methods given with the publicity attending the classes held at the bedside by great physicians and surgeons affords a guarantee that treatment shall be of the best, and of a nature to meet the strongest criticism. It is a fact, easy of demonstration, that the existence of a medical school in connection with a hospital is the greatest incentive to efficiency. All this is imperilled by the municipal idea. No community could exist without its medical schools, and the work must necessarily be the duty of the State or of the community as a whole as represented by the voluntary system. Medical education is not a local, but a national, nay, an imperial question, and so long as hospitals are necessary for the instruction and training of medical men, so long will the municipalisation of all hospitals be rendered an impossibility.

But there are many other ways of looking at the matter, so far as municipalisation is concerned. It is first of all necessary for those who insist upon everything being done for the people by the municipality at the expense of the ratepayers to prove that municipal hospitals have been successful. We could produce instances in this country at the present time proving that they are absolutely inefficient, and that the plan of making it everybody's business to look after the sick by entrusting even a necessary portion of this duty to a municipality ends in disaster, or leads to serious risks to the patients who may be unfortunate enough to be attacked by illness of a severe or repellant character. Again, they must prove that municipal hospitals can be made a saving and an advantage to the community; a saving in the sense that their administration is so satisfactory that it will far and away exceed the economy and efficiency of the voluntary system as we have it in England to-day. It is well known, and we will only mention two relatively recent and notorious instances—the Eastern Hospital scandal and the Brook Hospital extravagances—that where the money for hospitals is provided by the rates, there as matters stand at present you may continually have the gravest abuses, the grossest extravagances and the most unsatisfactory results.

We have merely dealt with this question to-day from one or two business aspects. Yet we have said enough to show that *primâ facie* it is not only not to the advantage of the community to attempt to municipalise all the hospitals, but that it is not even possible to do so if the welfare of the community is to be efficiently safeguarded. The whole English people will certainly unite to raise and uphold the voluntary system of hospitals. People may criticise the voluntary hospitals, but in effect they have produced the finest results of any system of hospitals the world has ever seen. Their financial position to-day is sufficiently strong to make that principle secure, so there is not only no justification, but little sense in discussing the municipalisation of hospitals. The proposal is chimerical, and merely calculated to mislead the unthinking. As a serious proposition it can never be entertained in practice by any old community which will take the trouble to thoroughly master the facts and understand the dangers and mischief which must attend its realisation.

